# Effects of insect longevity and drought conditions on aster leafhopper (Hemiptera: Cicadellidae) fecundity

**DOI:** 10.1093/ee/nvaf068

**Published:** 2025-07-02

**Authors:** Berenice Romero, Lawrence Entz, Christopher M Wallis, Sean M Prager

**Affiliations:** Department of Plant Sciences, College of Agriculture and Bioresources, University of Saskatchewan, Saskatoon, SK, Canada; Department of Plant Sciences, College of Agriculture and Bioresources, University of Saskatchewan, Saskatoon, SK, Canada; Crop Diseases, Pests and Genetics Research Unit, U.S. Department of Agriculture, Agricultural Research Service, San Joaquin Valley Agricultural Sciences Center, Parlier, CA, USA; Department of Plant Sciences, College of Agriculture and Bioresources, University of Saskatchewan, Saskatoon, SK, Canada

**Keywords:** Cicadellidae, fecundity, nutrition, age, offspring size

## Abstract

Insect population dynamics profoundly affect the potential for a species to serve as a pest, highlighting the importance of proper quantification and monitoring of insect reproduction. Important measurements of reproduction include individual female egg load and realized fecundity, which can be affected by insect longevity and host quality. Aster leafhoppers (*Macrosteles quadrilineatus* Forbes) are an important pest in Western Canada and the upper Midwest of the United States, yet little is known about factors influencing their fecundity, and thus, population dynamics. To evaluate age-specific changes in fecundity, newly emerged pairs of aster leafhoppers were caged onto plants, and egg and nymph numbers were determined on a weekly basis until females died. Moreover, water deficit can affect amino acid concentrations in phloem sap, and in turn, affect plant attractiveness and suitability for insect herbivores. To examine the relationship between water deficit and reproductive potential, aster leafhoppers were reared on unstressed and water-stressed barley plants until adult emergence. Pairs with all possible combinations of leafhoppers from each water stress condition were made and allowed to reproduce. Amino acid concentrations were quantified in unstressed and water-stressed barley plants. Aster leafhoppers produced eggs throughout their adult stage, with numbers decreasing as individuals got older. Females reared on water-stressed plants had fewer eggs following adult emergence. Following mating, females that had been reared on unstressed plants had a similar egg load to those that had been reared on water-stressed plants. Unstressed plants had a higher concentration of aspartic acid and a lower concentration of tryptophan.

## Introduction

The population dynamics of insects is of interest to several disciplines, particularly evolutionary ecology and agricultural entomology. In the context of agriculture, factors influencing population growth are of particular importance as population growth rates are essential to developing decision tools such as economic injury levels (Damos and Savopoulou-Soultani 2010, Chasen et al. 2015) and for predicting pest outbreaks (Hu et al. 2014, Ouyang et al. 2014). A key factor in population growth is fecundity, which can be defined in several ways but ultimately amounts to the number of viable offspring an individual female produces over the entire period of sexual maturity. In most insects, offspring are produced in the form of eggs and the lifetime fitness of a female insect is a function of the total number of eggs she successfully oviposits, and which develop to reproductive adults (Arnqvist and Nilsson 2000). This can, in turn, be influenced by both the total number of eggs she has available, the time she has to deposit the eggs, and offspring survival (Arnqvist and Nilsson 2000). Thus, female longevity and its effect on fecundity is an important factor driving insect population dynamics. In general, females with long lifespans will have the chance to lay more eggs throughout their lifetime and contribute a higher number of individuals to the next generation.

In some insects, the total number of eggs a female has available is fixed when they emerge as adults. In other species, eggs can continue to develop for some or all of the female’s lifespan (Jervis and Ferns 2004). In the former, one might expect the host quality of a juvenile’s host plant to influence the egg load of an herbivore as higher-quality hosts will provide more nutrition for developing eggs. In the latter, one might expect egg load to be influenced by the quality of the host the adult female feeds on (Awmack and Leather 2002, Jervis and Ferns 2004, Rosenheim et al. 2008). Consequently, a female’s ability to discriminate between suitable and unsuitable hosts, those that offer more or less nutrition, will play important roles in determining egg output and therefore potential fluctuations in population size (Jaenike 1978, Awmack and Leather 2002). Host quality can vary among plant species, among cultivars, or in response to different biotic and abiotic factors (Awmack and Leather 2002, Sisterson et al. 2015, 2017, Jamieson et al. 2017). In this context, abiotic factors such as drought can also affect the fecundity of phytophagous insects and thus their population dynamics (Jamieson et al. 2017). Earlier studies have shown that plants experiencing varying degrees of water stress will be often characterized by higher nitrogen levels or increased concentrations of certain amino acids (Hale et al. 2003, Seagraves et al. 2011,  Asiyeh Notghi Moghadam et al. 2022), and such changes to plant chemistry can either have a positive effect on insect nutrition and fecundity (Huberty and Denno 2004, Franzke and Reinhold 2011,  Asiyeh Notghi Moghadam et al. 2022) or be detrimental to insect development (Hale et al. 2003, Huberty and Denno 2004, Kansman et al. 2020).

Due to the confounding factors of longevity, egg load, and host quality, females often face a tradeoff between oviposition and their own nutritional requirements. In one scenario, they can abstain from oviposition while searching for higher-quality hosts at the potential cost of their own death or failure to find a host. Conversely, they can oviposit on an available lower-quality host reducing uncertainty (Awmack and Leather 2002, Rosenheim et al. 2008). Thus, one might expect older insects to be more likely to accept lower-quality hosts, provided they possess the ability to evaluate both host quality and its own internal condition. Furthermore, previous experience can additionally affect adults’ host choice selection behaviors (Olsson et al. 2006, Thöming et al. 2013).

Populations of aster leafhoppers (Hemiptera: Cicadellidae: *Macrosteles quadrilineatus* Forbes) are carried by wind currents into Western Canada and the upper Midwest of the United States each spring and early summer (Nichiporik 1965, Hoy et al. 1992, Frost et al. 2013). This migratory species is the primary vector of the bacterial plant pathogen Aster Yellows phytoplasma (Olivier et al. 2009, Bertaccini et al. 2019), which can infect over 300 different plant species and lead to outbreaks with devastating consequences in this region (Olivier et al. 2009, Frost et al. 2013, Romero et al. 2023). Despite recent work on the host choice selection and probing behaviors of aster leafhoppers (Romero et al. 2020, 2022, 2024a, 2024b), understanding how female longevity and host quality can affect aster leafhopper fecundity and reproductive output is paramount for developing more appropriate methods to monitor and predict changes in their population dynamics. Incorporating such information into pest forecasting models can improve their accuracy and utility, and thus, allow for earlier and more precise interventions (Nylin 2001, Crimmins et al. 2020, de Godoy et al. 2023).

In this study, we examined the relationship between aster leafhopper female longevity and fecundity by determining the number of eggs laid and the number of nymphs on a weekly basis and until each female died. We also conducted no-choice and two-choice bioassays with aster leafhoppers that had been reared on a water-stressed or an unstressed barley plant until adult emergence to investigate the relationship between host plant quality and aster leafhopper offspring numbers and host choice selection behavior. Further, the amino acid concentrations were quantified in both host plants to examine possible differences that could affect aster leafhopper biology. Based on previous work, we hypothesized that (i) aster leafhopper fecundity would decrease as females age, and (ii) water-stressed plants would be characterized by higher amino acid levels, and in turn, represent more suitable hosts for aster leafhopper development and egg production.

## Materials and Methods

### Plant Species and Growing Conditions

Plants for all experiments were grown according to procedures described by Romero et al. (2020, 2022), with a few modifications. Barley (Poales: Poaceae: *Hordeum vulgare* L., CDC Copeland) plants were grown at 21 °C during the day (16 h) and 18°C during the night (8 h). All plants were grown in round pots (10.2 cm diameter top, 7.4 cm diameter base, and 9 cm depth) filled with a soil mixture containing peat, perlite, and vermiculite (Sunshine Mix #4, Sun Gro Horticulture, Agawam, Massachusetts, United States). One to three barley seeds were planted in the center of each pot, at a depth of 2.50 cm. Following germination, additional seedlings were removed to ensure that each pot contained one plant. Water-soluble fertilizer with a nitrogen-phosphorous-potassium ratio of 20-20-20 (Miracle-Gro, Marysville, Ohio, United States) at a concentration of 0.350 g/L was used to meet the nutritional needs of the plants. Plants were watered every 3 d until emergence, after which they were divided into two groups (unstressed and water-stressed conditions) and watered accordingly. Field capacity can be defined as “the amount of water retained in the soil after the excess gravitational water has been drained away” (Veihmeyer and Hendrickson 1949). Unstressed conditions were achieved by maintaining the soil at field capacity, while the water-stressed conditions were created by maintaining the soil at half field capacity.

### Water Conditions

The field capacity of the plant growth media was determined experimentally. Flat bottom Falcon tubes of 50 ml volume were filled with 10 g of dry Sunshine Mix #1 soil mixture, to which different amounts of distilled water were added. The amount of water was expressed as a percentage of the total weight of the soil sample, and percentages between 25% (2.5 g) and 50% (5.0 g) of water were tested. The water was allowed to infiltrate the soil for 1 h, with the Falcon tubes maintained in an upright position. Field capacity was determined by examining the water volume that had complete penetration of the water to the bottom of the tube but experienced no pooling of excess water at the bottom. For Sunshine Mix #1, field capacity was found to be 47%. Field capacity of 47% soil moisture and half field capacity of 23.5% soil moisture were maintained using an electronic Flower Power plant sensor (Parrot, Paris, France). For the first 14 d of the experiment, soil moisture was checked and adjusted with the addition of water three times weekly. After 14 d, soil moisture was corrected to field capacity (unstressed conditions, ‘U’) and half field capacity (water-stressed conditions, ‘W’) daily. All plants were maintained at either field capacity or half field capacity for 1 wk prior to the introduction of the insects and continued until the completion of the experiments.

### Nutrient Compensation in Water-stressed Conditions

The fertilizer rate of plants subjected to half-field capacity conditions was adjusted to maintain equal nutrient levels between these plants and those from the field capacity conditions. To determine the total water volume needed for each treatment, two pots containing 50 g of dry Sunshine Mix #1 soil mixture each were taken, and water was added until the desired soil moisture level was reached. For the field capacity treatment, a rate of 0.350 g/L was used. To compensate for the 29% reduction in the water volume in the half-field capacity treatment, the rate of fertilizer was adjusted to 0.451 g/L. The total water volume needed to maintain field capacity and half field capacity conditions was measured at three growth stages of barley (BBHC 10, 21, and 37; Lancashire et al. 1991). At BBCH 10 and 21, the difference remained at 29%, which required using a fertilizer concentration of 0.451 g/L for the half-field capacity conditions. However, at BBCH 37, the difference in the water needed increased to 31%, and the fertilizer concentration for the half field capacity treatment was changed to 0.458 g/L for the remainder of the experiment.

### Aster Leafhoppers

Aster leafhoppers were reared as previously described by Romero et al. (2020, 2022). Insect colonies were maintained under an 18-h photoperiod, at 21 °C during the day and 18 °C during the night. Aster leafhoppers were reared on barley plants, which were replaced on a weekly basis. At any given time, more than one insect cohort and generation were present in the colonies. While this insect species can transmit a bacterial pathogen (*Candidatus* Phytoplasma asteris) to several plant species, only non-inoculative (uninfected) aster leafhoppers were used for the experiments in this study. Unless otherwise stated, only newly emerged (< 24 hs) adults were used.

### Fecundity Over Time and Female Survival

Barley plants were grown as previously described, under unstressed water conditions (field capacity). Three-week-old plants were used in all cases. Prior to the beginning of the experiments, fourth- and fifth-instar aster leafhoppers were collected from the colonies and placed on individual barley plants in groups of 10-15 insects. Insects were caged onto the plants using a similar cage to that described in Romero et al. (2020). Briefly, the cage was made from two clear plastic cups and an organza bag; this cage (referred to as ‘cup cage’) was inserted into the soil and allowed air flow in an out (Supplementary Fig. 1). Plants were observed daily for newly emerged adults, which were used to form mating groups consisting of one female and two males. Each mating group was caged onto a barley plant for one week using a cup cage, after which insects were placed on a new plant, and the old plant was either kept for conducting further observations (Group A) or stained to quantify the number of eggs within the plant tissues (Group B). This procedure was repeated for each mating groups until the female died, and additional males were occasionally added to each experimental unit to assure that two males were present at all times. Daily observations were conducted to assess female survival in all mating groups. For each group, 10 replicates were conducted. Plants in group A were maintained under the same environmental conditions and inspected for the presence of nymphs, while plants in group B were stained following procedures described by Romero et al. (2022). Briefly, shoot tissues were removed and placed in a Petri dish containing the McBride’s stain solution (95% of ethanol and glacial acetic acid -1:1 vol/vol- and 5% of 0.2% acid fuchsin; Commercial Alcohols and Fisher Chemical) for 24 h. Following this staining step, samples were transferred to new Petri dishes containing a clearing agent solution (Distilled water, glycerol, and lactic acid; 1:1:1 vol/vol/vol; Fisher Chemical). Samples were then incubated for 4 h at 75 °C and examined under a compound stereo microscope (Zeiss Stemi 305, Oberkochen, Germany).

By measuring the number of eggs in the plant tissues and the number of nymphs, we estimated the number of eggs that each female laid and their viability, both of which are important components of an insect’s reproductive output.

### No-choice Bioassays

To determine if the water conditions of the rearing host could affect the number of offspring of aster leafhoppers, mating pairs of aster leafhoppers reared under different water conditions (U and W) were formed. Three pairs of aster leafhoppers were caged onto a 3-wk-old barley plant for 1 wk, after which the insects were removed, and plants were kept for further observations. Females from the mating pairs were immediately stored in 70% ethanol and preserved for later dissection. There was a total of four breeding crosses (female U × male U; female W × male U; female U × male W; female W × male W), each of which was replicated 10 times. Additional newly emerged females reared on plants under unstressed and water-stressed conditions were collected and preserved for later dissection. In this case, between 54 and 67 females of each treatment were dissected. The individual female egg load was determined in each case, which represented the maximum possible number of eggs an insect could deposit prior to dissection. Plants were maintained under unstressed conditions until the completion of the experiment. Following the removal of the breeding pairs, plants were observed every 2 d for the presence of nymphs. The number and stage of nymphs were determined during each observation period. As the nymphs began to emerge as adults, they were removed from the plants and counted to confirm the total number that survived to adulthood.

### Two-choice Bioassays

For two-choice bioassays, procedures were like those described by Romero et al. (2022), with a few modifications. Newly emerged aster leafhoppers were sorted into groups of 8 males and 8 females based on external genitalia. All 16 leafhoppers were then released in a cage containing two test plants, which consisted of a barley plant of each water condition (U and W). Three-week-old plants were used. Insects were allowed to acclimate for 24 h, after which their position (plant 1, plant 2, or off the plant) was recorded daily for a total of 120 hs. Ten replicates were conducted for each insect group (insects reared on barley grown under U and insects reared on barley grown under W) and all observations were made between 9 and 11 am each day. After this, plants were kept for further processing and stained using a similar methodology to that described for Group B of the experiments to examine changes in female fecundity over time. Samples were examined under a compound stereo microscope (Zeiss Stemi 305, Oberkochen, Germany) and the number of salivary sheaths and eggs was determined in each case.

### Amino Acid Quantification

Additional barley plants grown under both water conditions (U and W) were selected for quantifying the amino acids present in the leaf tissues. Three-week-old plants were used. Procedures were similar to those described by Wallis et al. (2022). Briefly, leaf tissues from each plant were collected and pulverized in liquid nitrogen with a mortar and pestle. A total of 0.1 g of ground tissue was placed in a microcentrifuge tube. This tissue was then extracted twice in 0.5 ml of methanol (Fisher Scientific, Waltham, Massachusetts, US) at 4 °C overnight each time. From this methanol extract, 100 μl were used with a Phenomenex EX-FAAST amino acid kit for gas chromatography with a flame ionization detector (GC-FID) and a Phenomenex Zebron AA column. The amino acids were identified and quantified following the manufacturer’s protocol.

### Statistical Analysis

Statistical analyses were performed using R version 4.1.3 (R Core Team 2022). For the experiment to examine changes in aster leafhopper fecundity over time, results were analyzed using a Generalized Linear Mixed Model (GLMM) with a Poisson distribution, with “Week” as a fixed effect and the “individual female ID” as a random effect. For the individual female egg load of insects reared on field capacity or half field capacity plants, results were analyzed using a GLMM with a normal distribution, with “rearing host” as a fixed effect and the “date” on which the experiment was conducted as a random effect. For the individual female egg load of insects from each cross, results were analyzed using a GLMM with a Poisson distribution, with “male nutrition,” “female nutrition,” and their interaction as fixed effects. In this model, the “date” on which the experiment was conducted was incorporated as a random effect. The total number of individuals, the total number of nymphs, and the total number of adults were analyzed using a GLMM with a negative binomial distribution, with “female nutrition,” “male nutrition,” and their interaction as fixed effects. In these models, the “date” on which the experiment was incorporated into each model as a random effect. The package “lme4” (Bates et al. 2015) was used to conduct the analyses and the package “emmeans” (Russell 2022) was used to perform post-hoc tests. In the two-choice bioassays, the number of aster leafhoppers on each plant was analyzed using a permutational multivariate analysis of variance (PERMANOVA; Oksanen et al. 2022). In each PERMANOVA analysis, the distance matrix was composed of the number of leafhoppers observed on the plants (U or W) each day (day 1, day 2, day 3, or day 4), and the “choice plant” was included as the explanatory variable. The numbers of salivary sheaths and eggs were analyzed with a paired *t*-test for each combination offered during the two-choice bioassay. When residuals were not normally distributed, the Wilcoxon test was used instead. Amino acid levels were analyzed using individual GLMMs with a normal distribution, with “rearing host” as a fixed effect and the “date” of the extraction as a random effect. *P*-values were then adjusted using the Bonferroni correction for multiple testing.

## Results

### Female Survival and Effects of Insect Longevity on Fecundity

**Fig. 1.**
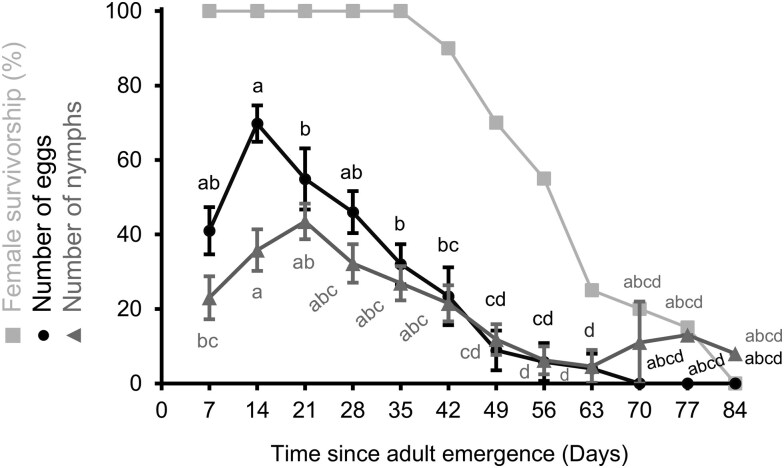
Survivorship (light gray square, ■), number of eggs (black circle, ●), and number of nymphs (dark gray triangle, ▲) of aster leafhopper females. The survivorship was assessed daily, and the numbers of eggs and nymphs produced were determined on a weekly basis. For the numbers of eggs and nymphs, the mean and standard error of the mean (SEM) values have been provided. The numbers of eggs displayed are corrected for the number of surviving females. Different letters indicate statistically significant differences in the number of eggs and nymphs observed each week (GLMM followed by Tukey’s test with adjustment for multiple comparisons, with an α-value of 0.05).

To examine the relationship between female longevity and fecundity, newly emerged aster leafhopper females were caged onto unstressed barley plants, and their egg and nymph production was determined each week until all individuals died. This experimental setup also allowed to track the survivorship of all females. Aster leafhopper females lived between 35 and 84 d, and their fecundity gradually decreased over time (Fig. 1). Each female laid an average of 278.1 ± 27.1 eggs and produced a mean of 208.4 ± 20.8 nymphs during their adult lifespan, indicating that the overall hatching rate was approximately 74.9%. We found a significant effect of time on the number of eggs present in leaf tissues (χ^2^ = 123.080, df = 11, *P* < 0.001) and on the number of nymphs observed (χ^2^ = 74.569, df = 11, *P* < 0.001) (Fig. 1). In both cases, there was a trend by which the numbers of eggs and nymphs seemed to increase as females reach an age of 14 to 21 d and then gradually decreased as females got older (Fig. 1).

### Effects of Water Conditions on Fecundity

**Fig. 2.**
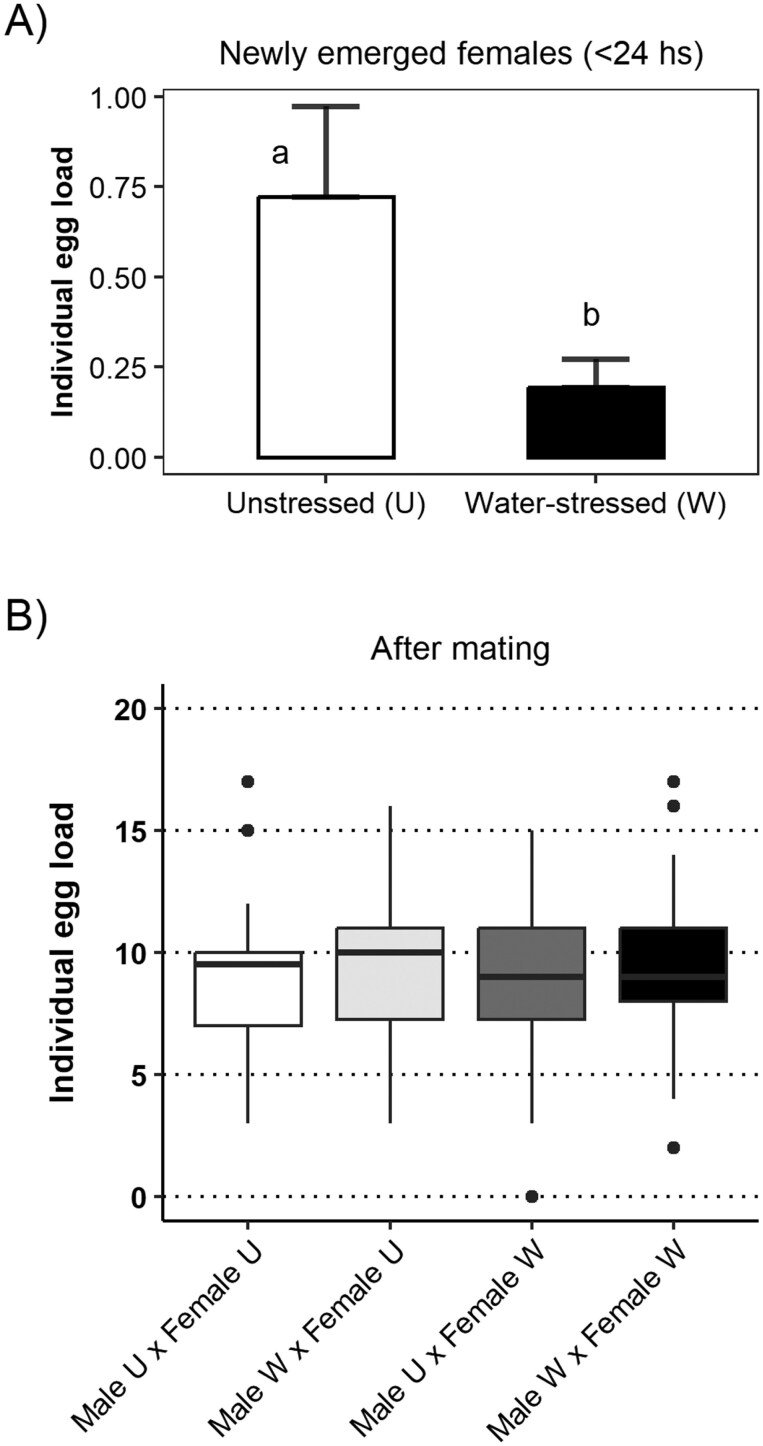
Individual egg load of newly emerged females (< 24 hs) (A) and newly emerged females following their mating period with males from different treatments (B). Insects reared on unstressed barley plants have been indicated with a U, while insects reared on water-stressed barley plants have been indicated with a W. A) For the individual egg load of newly emerged females, the bars represent the mean values, and the whiskers indicate the SEM. Different letters indicate statistically significant differences in the individual egg load of females from both treatments. B) For the egg load of females following their mating period, boxes are drawn between the 25th and 75th percentiles, with the median marked with a horizontal black line. Whiskers indicate the largest and smallest values within 1.5 times interquartile range from the ends of the boxes.

To examine the relationship between insect nutrition and fecundity, groups of aster leafhoppers were reared on unstressed (U) and water-stressed (W) barley plants. We found a significant effect of the rearing host on the individual female egg load of newly emerged aster leafhoppers (χ^2^ = 16.864, df = 1, *P* < 0.001), with females reared on unstressed plants having a higher egg load (0.72 ± 025, Mean ± SEM) than those reared on water-stressed plants (0.19 ± 0.08) (Fig. 2A).

Additional newly emerged females were selected to form mating pairs with newly emerged males from both rearing hosts. Following their mating period of 1 wk, all adults were removed from the plants, and the individual egg load of all females was determined (Fig. 2B). We found no significant effect of the male nutrition (χ^2^ = 0.131, df = 1, *P* = 0.717), female nutrition (χ^2^ = 0.091, df = 1, *P* = 0.763), and their 2-way interaction (χ^2^ = 0.014, df = 1, *P* = 0.907) on the individual egg load of aster leafhopper females (Fig. 2B). Females from the different treatments had between 9.00 ± 0.63 (Male U × Female W) and 9.37 ± 0.56 eggs (Male W × Female U) within their reproductive tracts following their mating period (Fig. 2B).

**Fig. 3.**
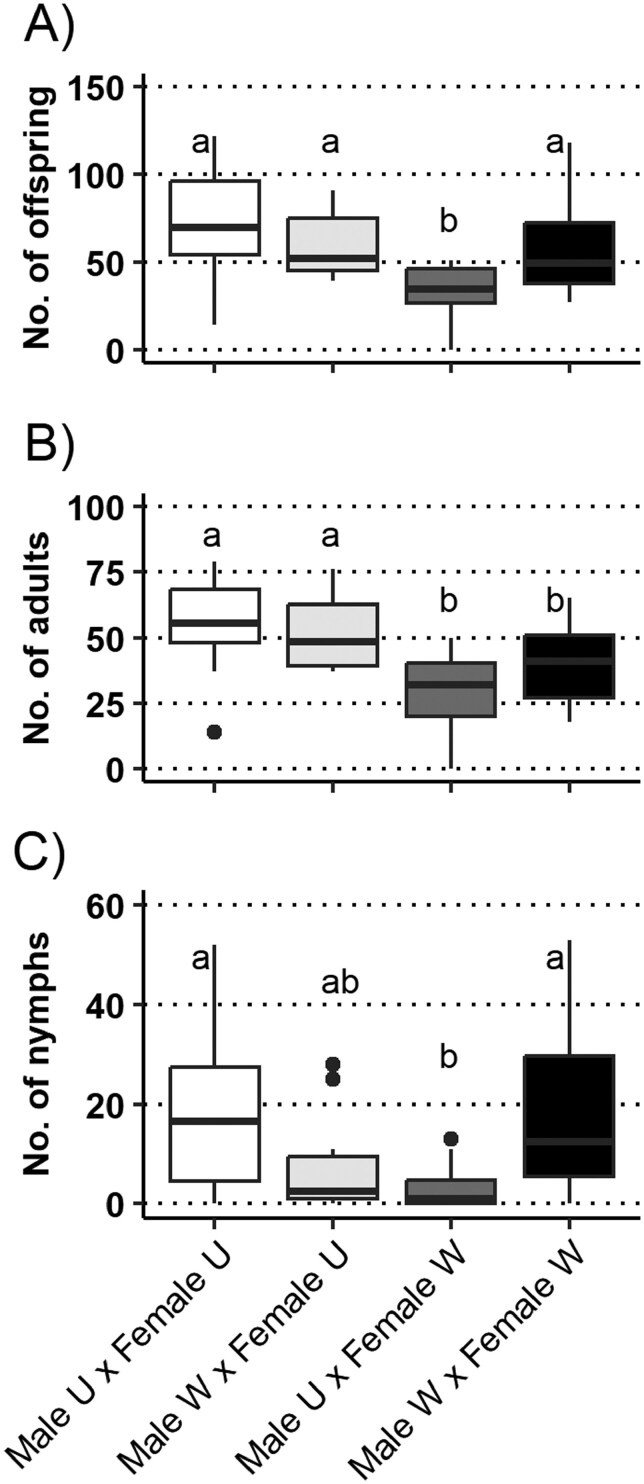
Number of aster leafhopper offspring for all mating crosses between newly emerged adults from unstressed (U) and water-stressed (W) plants. For additional analyses, offspring numbers were separated into adults (B) and nymphs (C). Boxes are drawn between the 25th and 75th percentiles, with the median marked with a horizontal black line. Whiskers indicate the largest and smallest values within 1.5 times interquartile range from the ends of the boxes. Different letters indicate statistically significant differences among treatments (GLMM followed by Bonferroni correction for multiple comparisons, with an α-value of 0.05).

Following the mating period, pairs of aster leafhoppers were removed and plants were kept for further examination. We found no significant effect of the male nutrition (χ^2^ = 1.504, df = 1, *P* = 0.220) on the number of offspring of aster leafhoppers, but there was a significant effect of the female nutrition (χ^2^ = 5.814, df = 1, *P* = 0.015) and the 2-way interaction (χ^2^ = 6.207, df = 1, *P* = 0.013) on this variable (Fig. 3A). While most mating pairs were associated with offspring numbers ranging between 59.60 ± 5.67 (Male W × Female U) and 72.10 ± 10.30 (Male U × Female U), mating pairs comprised by males from unstressed plants and females from water-stressed plants had fewer offspring (33.50 ± 5.16; Fig. 3A).

Offspring numbers were additionally separated into adults (Fig. 3B) and nymphs (Fig. 3C) for further analysis. We found no significant effect of the male nutrition (χ^2^ = 0.615, df = 1, *P* = 0.433) or 2-way interaction (χ^2^ = 1.257, df = 1, *P* = 0.262) on the number of offspring that reached the adult stage, but there was a significant effect of female nutrition (χ^2^ = 9.490, df = 1, *P* = 0.002) on this variable (Fig. 3B). Mating pairs with females from unstressed plants were associated with greater numbers of offspring that reached the adult stage (52.00 ± 4.80 in Male W × Female U and 54.30 ± 6.10 in Male U × Female U) compared to treatments with females from water-stressed plants (30.00 ± 5.02 in Male U × Female W and 39.40 ± 5.07 in Male W × Female W) (Fig. 3B). Analyses of the number of nymphs revealed no significant effect of the male nutrition (χ^2^ = 1.077, df = 1, *P* = 0.299) or female nutrition (χ^2^ = 0.451, df = 1, *P* = 0.502), but a significant effect of the 2-way interaction (χ^2^ = 9.783, df = 1, *P* = 0.002) (Fig. 3C). Most mating pairs were associated with nymph numbers ranging between 7.60 ± 3.33 (Male W × Female U) and 20.70 ± 6.26 (Male W × Female W), except for those involving males reared on unstressed plants and females reared on water-stressed plants, which were characterized by a mean of 3.50 ± 1.53 nymphs per plant (Fig. 3C).

### Effects of Water Conditions on Settling, Oviposition, and Probing Behaviors

**Fig. 4.**
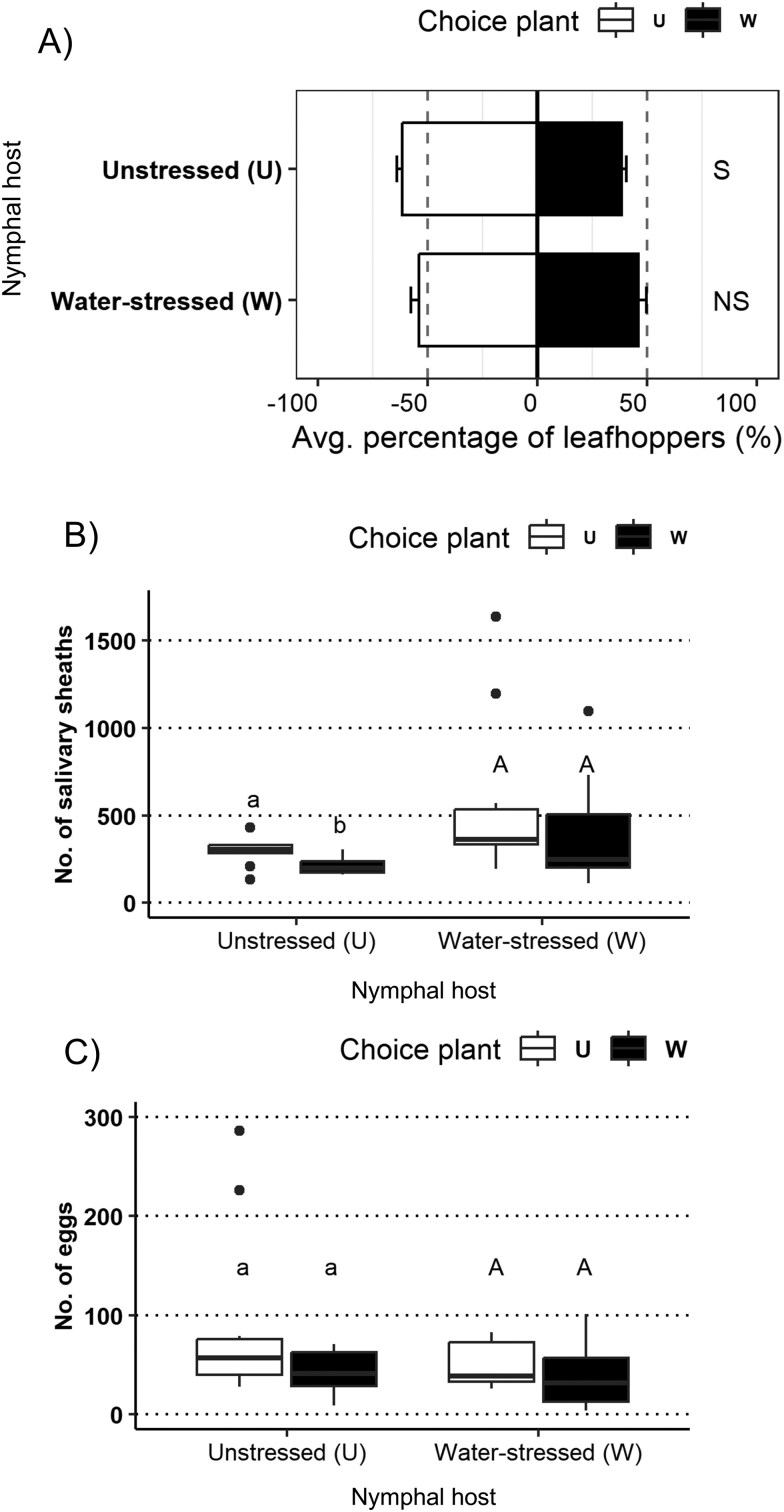
A) Settling behavior of groups of newly emerged aster leafhoppers reared on unstressed (U) and water-stressed (W) barley plants. Choice plants included a pot containing a U barley plant and another pot containing a W barley plant. Bars represent the avg. percentage (Mean ± SEM) of leafhoppers on each plant for each plant combination for the entire observation period (120 hs). Settling behavior results were evaluated using a PERMANOVA analysis, with a significance level (α-value) of 0.05. ‘S’ represents a plant combination in which a settling preference was observed, while ‘NS’ indicates that aster leafhoppers distributed similarly on both test plants provided. B) The number of salivary sheaths was used as proxy for feeding activity and results were evaluated using a paired t-test for each combination. If residuals were not normally distributed, the Wilcoxon test was used instead. Different letters indicate statistically significant differences among treatments (α-value of 0.05). C) Oviposition event results were evaluated using a paired t-test for each combination. If residuals were not normally distributed, the Wilcoxon test was used instead.

Groups of aster leafhoppers reared on unstressed and water-stressed barley plants were provided with two choice plants to assess their settling preferences (Fig. 4A). Aster leafhoppers that had been reared on unstressed plants exhibited a preference for settling on plants with similar characteristics to their rearing host (PERMANOVA, *P* = 0.001), with 61.60 ± 2.30% of the insects settling on unstressed plants and 38.40 ± 2.30% on water-stressed plants (Fig. 4A). However, insects that had been reared on water-stressed plants exhibited no preference for settling on either plant in the combination (PERMANOVA, *P* = 0.063), with 54.06 ± 3.65% of the insects settling on unstressed plants and 45.94 ± 3.65% on water-stressed plants (Fig. 4A). Following the two-choice bioassays, the numbers of eggs and salivary sheaths in leaf tissues were determined (Fig. 4B–C). Differences were observed in the number of salivary sheaths in plants that had been exposed to aster leafhoppers reared on unstressed plants (paired *t*-test, *P* = 0.007), with water-stressed plants having fewer salivary sheaths (209.70 ± 14.56) than unstressed plants (293.50 ± 24.87) (Fig. 4B, left). Plants that had been exposed to insects reared on water-stressed plants had a similar number of salivary sheaths (Wilcoxon test, *P* = 0.557), with 570.0 ± 148.04 salivary sheaths on unstressed plants and 389.30 ± 99.36 on water-stressed plants (Fig. 4B, right). Examination of stained plant tissues also revealed that both insect groups laid a similar number of eggs on both plants offered during the two-choice bioassays (Wilcoxon test, *P* = 0.432 for U aster leafhoppers; paired *t*-test, *P* = 0.966 for W aster leafhoppers). Plants that had been exposed to insects reared on unstressed plants had between 91.30 ± 28.27 (U) and 42.70 ± 6.82 (W) eggs (Fig. 4C, left), while plants that had been exposed to insects reared on water-stressed plants were characterized by egg numbers ranging between 50.10 ± 7.10 (U) and 39.10 ± 10.32 (W) (Fig. 4C, right).

### Amino Acid Levels in Unstressed and Water-stressed Plants

To examine the relationship between aster leafhopper behavioral responses and host quality, the levels of amino acids in leaf tissues were determined for both unstressed and water-stressed plants (Table 1). In most cases, unstressed and water-stressed plants had similar levels of amino acids (Table 1). However, the levels of aspartic acid and tryptophan differed between these plants. Unstressed plants were characterized by a higher concentration of aspartic acid compared to water-stressed plants (1.433 ± 0.211 μmol/g FW in unstressed plants vs. 0.899 ± 0.044 μmol/g FW in water-stressed plants), while water-stressed plants contained a higher concentration of tryptophan compared to unstressed plants (0.028 ± 0.004 μmol/g FW in unstressed plants vs. 0.067 ± 0.016 μmol/g FW in water-stressed plants) (Table 1).

**Table 1:** Amino acid levels (μmol/g FW) present in leaves of unstressed (U) and water-stressed (W) barley plants. Mean (and SEM) values are provided. P-values shown in bold indicate cases in which amino acid levels differ between U and W plants (Individual GLMM followed by Bonferroni correction for multiple testing, with an α-value of 0.05).

	Concentration (μmol/g FW) (Mean ± SEM)		
	Unstressed barley (U)	Water-stressed barley (W)	Adjusted P-value
Alanine	1.622 ± 0.186	1.346 ± 0.078	1.000
Asparagine	0.759 ± 0.088	1.178 ± 0.302	1.000
**Aspartic acid**	**1.433 ± 0.211**	**0.899 ± 0.044**	**0.004**
Glutamic acid	0.413 ± 0.082	0.231 ± 0.048	0.700
Glutamine	1.106 ± 0.303	0.846 ± 0.241	1.000
Glycine	0.349 ± 0.072	0.259 ± 0.059	0.703
Histidine	0.063 ± 0.003	0.083 ± 0.013	1.000
Isoleucine	0.273 ± 0.026	0.367 ± 0.081	1.000
Leucine	0.418 ± 0.037	0.454 ± 0.054	1.000
Lysine	0.333 ± 0.047	0.304 ± 0.021	1.000
Methionine	0.104 ± 0.008	0.080 ± 0.001	0.361
Phenylalanine	0.244 ± 0.028	0.274 ± 0.043	1.000
Proline	0.098 ± 0.018	1.558 ± 0.607	0.090
Serine	5.920 ± 1.408	5.037 ± 0.960	1.000
Threonine	0.534 ± 0.116	0.437 ± 0.105	0.401
**Triptophan**	**0.028 ± 0.004**	**0.067 ± 0.016**	**0.032**
Tyrosine	0.128 ± 0.018	0.122 ± 0.012	1.000
Valine	0.536 ± 0.036	0.641 ± 0.082	1.000

## Discussion

The relationship between female fecundity and female age at the time of mating has been characterized for several insect species with different breeding strategies and host characteristics (Chi and Su 2006, Stahl et al. 2019, Rodríguez-Gómez et al. 2022, Straser and Wilson 2022, Ali et al. 2023). Similarly, there is an extensive body of literature about the effects of parental nutrition on reproduction, fecundity, and offspring development (Miller et al. 2014, May et al. 2015, Benelli et al. 2017, Dihn et al. 2021). While most of this work has focused on model organisms such as *Drosophila* spp. or on hymenopteran species due to their potential to control pest populations, it is paramount to extend these approaches and research questions to other insect groups. Some members within the order Hemiptera are responsible for the transmission of approximately 55% of plant viruses (Hogenhout et al. 2008) and other plant pathogens like phytoplasmas (Weintraub and Beanland 2006, Eigenbrode et al. 2017). A comprehensive study of the biology of these insect vectors, including factors exerting changes in their fecundity and offspring size, would help in setting a framework for comparative information and to inform and develop more effective management practices to control their populations. For these reasons, we examined the effects of female age and parental nutrition on aster leafhopper fecundity and offspring numbers.

Aster leafhopper females were observed to lay fewer eggs within their first week as adults compared to the number of eggs laid in the following week, after which the number of eggs gradually decreased over time. This pattern possibly suggests that newly eclosed aster leafhoppers require a few days for sexual maturation to occur and that following this period, eggs are produced and matured throughout the remainder of their adult stage. Previous work with different members in the Pentatomidae family indicated that 5 to 17 d are required for these insects to reach sexual maturity and begin mating and that mating events decline as females get older (Mitchell and Mau 1969, Phelan and Frumhoff 1991, Ho and Millar 2001). Similar trends can also be seen in more distant insect species such as *Bactrocera tryoni* Froggatt (Diptera: Tephritidae), which requires approximately 12 d to become sexually mature and produce eggs, and their fecundity has been observed to decrease over time (Tasnin et al. 2021). Our study also indicated that aster leafhopper female adults have a lifespan of 42 to 84 d, which is consistent with previous observations for other hemipteran species (Phelan and Frumhoff 1991, Rodríguez-Gómez et al. 2022). Phelan and Frumhoff (1991) reported that two species of *Oncopeltus* sp. (Hemiptera: Lygaeidae) had a lifespan ranging between 78 and 87 d, while Rodríguez-Gómez et al. (2022) observed that female adults of *Orius laevigatus* Fieber (Hemiptera: Anthocoridae) were characterized by a lifespan of 47 to 60 d.

Female survivorship and fecundity can be influenced by different factors, including environmental conditions, diet characteristics, mating status, and the age of the female (Wittmeyer et al. 2001, Lee et al. 2008, Papanastasiou et al. 2013, Arganda et al. 2017, Tasnin et al. 2021, Cirino et al. 2022, Rodríguez-Gómez et al. 2022). In our study, newly emerged aster leafhopper females that had been reared on unstressed barley plants had a higher egg load than those reared on water-stressed barley plants, suggesting that host plant quality and developmental nutrition can affect certain life-history traits in this insect species. These differences between aster leafhopper females, however, were no longer observed following their exposure to unstressed barley plants during the mating period. One explanation for this could be that aster leafhopper female adults can recover from the effects of poor nutrition during their nymphal stage, which is consistent with previous observations for other insect species such as *Podisus maculiventris* Say (Hemiptera: Pentatomidae) (Wittmeyer et al. 2001), *Tribolium castaneum* Herbst (Coleoptera: Tenebrionidae) (Plesnar-Bielak et al. 2017), *Narnia femorata* Stål (Hemiptera: Coreidae) (Cirino et al. 2022), and *Homalodisca vitripennis* Germar (Hemiptera: Cicadellidae) (Sisterson et al. 2017). Cirino et al. (2022) examined the relationship between host quality and life history traits of N. *femorata* using unripe cactus fruits (low-quality), ripe cactus fruits (high-quality), or a combination of both. These authors reported that *N. femorata* females feeding on unripe cactus fruits during the nymphal stage followed by ripe cactus fruits during the adult stage had a higher survival and fecundity than those insects reared exclusively on unripe fruits (Cirino et al. 2022). Survival and fecundity in females receiving the combination of unripe and ripe cactus fruits, however, were lower when compared to insects reared exclusively on ripe fruits, indicating that the recovery from the poor nutrition received during their nymphal stage was only partial (Cirino et al. 2022). Similar findings were reported by Sisterson et al. (2017), who used a combination of high-quality and low-quality hosts to examine their effects of the egg maturation of *H. vitripennis*.

Parental nutrition can affect population dynamics by exerting changes on female reproduction success, egg viability, and offspring life traits such as developmental time and growth rate (Delisle and Bouchard 1995, Delisle and Hardy 1997, Frago and Bauce 2014 , Gutiérrez et al. 2020, Macartney et al. 2022). In this study, mating pairs in which both parents had been reared under the same conditions produced a similar number of offspring, yet those composed of males that had been reared on unstressed hosts and females reared on water-stressed hosts (Male U × Female W) were observed to have lower offspring numbers. These results suggest a complex interaction among parental male nutrition, parental female nutrition, and offspring nutrition, which has been documented in previous work with other insect species such as *Choristoneura fumiferana* Clemens (Lepidoptera: Tortricidae) and *C. rosaceana* Harris (Lepidoptera: Tortricidae) (Frago and Baucé 2019, Macartney et al. 2022).

While aster leafhoppers that had been reared on an unstressed barley plant preferred to settle and probe on a host with similar characteristics, insects that had been reared on water-stressed barley plants exhibited no preference for settling or probing on either option provided. Previous work with this insect species had indicated that behavioral responses such as settling preferences were not affected by the rearing host, as the settling behavior of aster leafhoppers reared on barley plants was similar to that of insects reared on fleabane plants (Romero et al. 2022). These findings are in contrast with those of Stockon et al. (2016), who reported that *Diaphorina citri* Kuwayama (Hemiptera: Psyllidae) females exhibited a preference for the plant on which they had developed on as nymphs. This preference, however, later changed to match the plant species to which *D. citri* adults had been more recently exposed to (Stockon et al. 2016), suggesting that adult experiences can also influence host choice selection behavior. Initial work with cicadellids by  Saxena and Saxena (1974)   indicated that the physiological state of the insects could affect their behavioral responses, observing differences in the behavior of desiccated leafhoppers compared to that of water-satiated insects. Moreover, work on the effects of water limitation on plant-insect interactions has shown that changes in nitrogen availability, phloem sap viscosity, and turgor pressure can negatively affect the ability with which sucking-piercing insects access resources present in the phloem tissues, thus reducing their performance and altering their physiological state (Oswald and Brewer 1997, Hale et al. 2003, Huberty and Denno 2004, Kansman et al. 2020, 2022). Therefore, it is possible that the lack of preference observed in aster leafhoppers reared on water-stressed barley plants could be associated with an altered physiological state compared to insects reared on control plants.

Nutritional requirements vary across insect species and life stages ( Schoonhoven et al. 2005), and changes in the diet’s amino acid content can have various effects on insect development, survival, and reproduction (Koyama 1985, Brodbeck and Strong 1987, Pan et al. 2014, Sisterson et al. 2017, Moriyama and Fukatsu 2022, Tierney et al. 2023). In this study, water-stressed barley plants were a less optimal nutritional source for newly emerged leafhoppers and an overall less preferred host. Additionally, these plants were characterized by higher concentrations of tryptophan and lower concentrations of aspartic acid. In previous studies with *Empoasca fabae* Harris (Hemiptera: Cicadellidae), Hoffmann and Hogg (1991, 1992) and Hoffmann et al. (1991) reported that *E. fabae* preferred to settle on and oviposit on well-watered alfalfa plants, which were characterized by higher nitrogen levels compared to plants with moderate or severe water stress. Similarly, Brodbeck et al. (1990, 1996, 2004) observed that different plant species with higher concentrations of glutamine and asparagine were more suitable hosts for *Homalodisca coagulata* Say (Hemiptera: Cicadellidae) settling and development. Based on these observations, it is possible that the nutrition provided by barley plants with a lower concentration of aspartic acid could have negatively affected aster leafhopper egg production and physiology, yet additional experiments would be required to further support this hypothesis.

The aims of this study were to examine factors influencing fecundity in aster leafhoppers such as host quality and insect longevity, and to determine if rearing host characteristics can affect leafhopper behavioral responses. Our findings show that aster leafhopper females can produce eggs throughout their adult stage, with the number of eggs decreasing as these individuals age but the egg-hatching rate remaining constant. Host quality was also observed to affect aster leafhopper egg production and settling preferences, and individuals reared on a suboptimal host during their nymphal stage were observed to recover when allowed to feed on a more suitable host. These findings suggest that water stress in plants can have a negative impact on leafhopper reproduction and thus population dynamics and phytoplasma transmission, yet access to a more optimal host for a brief period of time can help leafhoppers recover from this. Previous work on different plant pathosystems has demonstrated that insect infection with a pathogen can have positive, neutral, or negative effects on insect survivorship, longevity, fecundity, and feeding behavior (Murral et al. 1996, Beanland et al. 2000, Bressan et al. 2005, Stafford et al. 2011, Nachappa et al. 2014, 2016, Arismendi and Carrillo 2015, Lei et al. 2016). This, in combination with drought-mediated plant-insect interactions, adds additional complexity and limits our ability to accurately predict if water-stressed plants are more or less susceptible to infection with any given pathogen (Szczepaniec1and Finke, 2019). In a recent study by Nachappa et al. (2016), drought-stressed soybean plants were observed to sustain smaller populations of an insect vector and lower levels of infection compared to saturated plants, and it would be possible for something similar to happen with phytoplasma-infected aster leafhoppers and water-stressed barley plants. However, additional research would be required to investigate such relationship between drought and phytoplasma disease dynamics. Additionally, development of aster leafhopper nymphs on suboptimal hosts can affect their host choice selection behavior, which could also influence population dynamics depending on the availability of other host plants nearby. It should be noted that while the concentration of two amino acids differed between hosts, additional experiments in which the concentrations of amino acids and other compounds are modified in artificial diets (Hou and Lin 1979, Koyama 1985, Pan et al. 2014) as well as studies using different plant varieties or plant species with contrasting chemical profiles (Brodbeck et al. 1990, 2004) are needed to increase our understanding of the impact of these compounds on aster leafhopper biology.

## Supplementary Material

nvaf068_Supplementary_Figure_S1

## Data Availability

The data that support the findings of this study are openly available in Open Science Framework (OSF) at https://doi.org/10.17605/OSF.IO/DKSP3.
