# Supplementary figures and images for: Effects of insect longevity and drought conditions on aster leafhopper (Hemiptera: Cicadellidae) fecundity
Source: Environ Entomol. 2025 Jul 2;54(5):1077–87. doi: 10.1093/ee/nvaf068 (PMC12543321; doi:10.1093/ee/nvaf068)

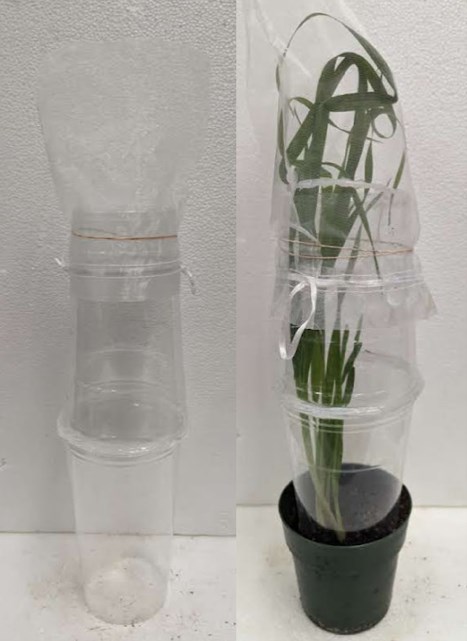

Supplement: nvaf068_Supplementary_Figure_S1 [file nvaf068_supplementary_figure_s1.jpeg]
